# Activation of Invariant Natural Killer T Cells Redirects the Inflammatory Response in Neonatal Sepsis

**DOI:** 10.3389/fimmu.2018.00833

**Published:** 2018-04-23

**Authors:** Alexandra C. Bolognese, Weng-Lang Yang, Laura W. Hansen, Archna Sharma, Jeffrey M. Nicastro, Gene F. Coppa, Ping Wang

**Affiliations:** ^1^Elmezzi Graduate School of Molecular Medicine, Manhasset, NY, United States; ^2^Department of Surgery, Zucker School of Medicine at Hofstra/Northwell, Hempstead, NY, United States; ^3^Center for Immunology and Inflammation, The Feinstein Institute for Medical Research, Manhasset, NY, United States

**Keywords:** neonatal sepsis, invariant natural killer T cells, KRN7000, CD1d, inflammation, lung injury, IFN-γ, TGF-β1

## Abstract

Sepsis is the third leading cause of death in the neonatal population, due to susceptibility to infection conferred by immaturity of both the innate and adaptive components of the immune system. Invariant natural killer T (iNKT) cells are specialized adaptive immune cells that possess important innate-like characteristics and have not yet been well-studied in septic neonates. We hypothesized that iNKT cells would play an important role in mediating the neonatal immune response to sepsis. To study this, we subjected 5- to 7-day-old neonatal C57BL/6 mice to sepsis by intraperitoneal (i.p.) cecal slurry (CS) injection. Thirty hours prior to or immediately following sepsis induction, pups received i.p. injection of the iNKT stimulator KRN7000 (KRN, 0.2 µg/g) or vehicle. Ten hours after CS injection, blood and tissues were collected for various analyses. Thirty-hour pretreatment with KRN resulted in better outcomes in inflammation, lung injury, and survival, while immediate treatment with KRN resulted in worse outcomes compared to vehicle treatment. We further analyzed the activation status of neonatal iNKT cells for 30 h after KRN administration, and showed a peak in frequency of CD69 expression on iNKT cells and serum IFN-γ levels at 5 and 10 h, respectively. We then used CD1d knockout neonatal mice to demonstrate that KRN acts through the major histocompatibility complex-like molecule CD1d to improve outcomes in neonatal sepsis. Finally, we identified that KRN pretreatment exerts its protective effect by increasing systemic levels of TGF-β1. These findings support the importance of iNKT cells for prophylactic immunomodulation in neonates susceptible to sepsis.

## Introduction

Sepsis is defined as a dysregulated host response to infection that results in life-threatening organ dysfunction (1). Sepsis remains a leading cause of morbidity and mortality worldwide, with peak incidences at the extremes of age. In fact, sepsis is the third leading cause of death during the neonatal period, from birth to 28 days of life (2–4). The prevalence of sepsis among very low birth weight (<1,500 g) neonates is considerably high with estimates of 25–30% and mortality estimates as high as 52% (5). In the United States alone, neonatal infections account for an estimated 40,000 to 50,000 deaths per year along with a financial burden of almost $700 million (6).

The immune system serves as the body’s major defense against infection. However, over-stimulation of immune cells during sepsis causes hyperinflammation that leads to organ injury and dysfunction (7). In addition, subsequent immunosuppression seen in sepsis predisposes patients to the development of secondary infections (8). By better understanding the response profiles of various immune cells during sepsis, we will be better able to target their responses and prevent sequelae including end-organ failure and death. Natural killer T (NKT) cells are members of the innate-like lymphocyte (ILL) class of immune cells (9). ILLs are cells that are characterized as members of the adaptive immune system by virtue of possessing a B or T cell receptor, but which also possess characteristics that they share with many cells of the innate immune system (10, 11). These characteristics make ILLs available as a first line of defense in infection, unlike conventional cells of the adaptive immune system, which require recruitment and clonal expansion over the course of days. Given the immaturity of the innate and adaptive immune responses in neonatal sepsis, there may be an important role for such cells that straddle the line between the two conventionally defined systems. Invariant natural killer T (iNKT) cells are the major subset of NKT cells and are so named because they possess an invariant T cell receptor α chain (Vα14Jα18 in mice and Vα24Jα18 in humans) combined with a limited number of β chains (Vβ8.2, Vβ7, or Vβ2 in mice and Vβ11 in humans) (12, 13). These cells require the major histocompatibility complex-like molecule CD1d for development and respond with specificity to glycolipid antigens presented by this molecule (14, 15). In adults, iNKT cells behave in an innate fashion in response to danger signals, increasing in number and rapidly producing a broad range of cytokines, most notably interferon (IFN)-γ and interleukin (IL)-4, within minutes to hours of activation (12, 13, 15). iNKT cells may also be stimulated directly through toll-like receptor (TLR) engagement, leading to enhanced IFN-γ but reduced IL-4 production (16). Activated iNKT cells affect myriad downstream immune responses, with direct and/or indirect effects on NK and B cell activation, T cell activation and differentiation, neutrophil recruitment, macrophage activation and polarization, and dendritic cell activation and cross-presentation (13).

The first identified lipid antigen of iNKT cells was α-galactosylceramide (α-GalCer) (13, 17). This discovery paved the way for the development of α-GalCer-loaded CD1d tetramers, facilitating the identification of iNKT cells using flow cytometry. In the same line, KRN7000, a synthetic α-GalCer analog, has been demonstrated to effectively activate iNKT cells in adult mice, and it has been reported to have potential therapeutic effects. For example, administration of α-GalCer after the creation of myocardial infarction in mice leads to an increase in survival rate (18). KRN7000 has additionally been shown to prevent graft-vs.-host disease in an adult mouse model (19).

To date, there has been little study of iNKT cells in neonates, and especially of their role in sepsis. Given our current understanding of the immune response to sepsis, and the capacity of adult iNKT cells to effect downstream immune functions, we hypothesized that iNKT cells would play an important role in mediating the immune response in neonatal sepsis. In this study, we employed a polymicrobial sepsis mouse model using intraperitoneal (i.p.) cecal slurry (CS) injection to determine the effect of activation of iNKT cells by KRN7000 at different time points on inflammation, lung injury, and survival in neonates. We then monitored the dynamic change of hepatic iNKT cells in healthy neonates after stimulation with KRN7000. We further used CD1d knockout (KO) mice to validate CD1d’s mediation of the effect of KRN7000. Finally, we used a cytokine array to identify that transforming growth factor (TGF)-β1 is a downstream product of iNKT cell activation, and further characterized the effect of TGF-β1 treatment in septic neonatal mice.

## Materials and Methods

### Experimental Animals

Neonatal mice were defined as those 5–7 days old. C57BL/6 male and female neonatal mice were obtained from in-house breeder pairs or from pregnant dams purchased from Charles River Laboratories (Wilmington, MA, USA). B6.129S6-Del(3Cd1d2-CD1d1)1Sbp/J (CD1d KO) breeder pairs were purchased from The Jackson Laboratory (Bar Harbor, ME, USA). A CD1d KO colony was established and neonatal mice were then obtained from the in-house breeder pairs. For all experiments pups were litter- and weight-matched between groups. Neonatal mice were housed with their mothers and breast-fed *ad libitum* before and after all experimental procedures. In-house young adult male and female C57BL/6 mice (20–25 g) were used for CS preparation. All experiments were performed in accordance with the guidelines for the use of experimental animals by NIH (Bethesda, MD, USA), and were approved by the Institutional Animal Care and Use Committee of the Feinstein Institute for Medical Research. All mice were housed in a temperature-controlled room with a 12-h light–dark cycle. For all *in vivo* experiments, anesthesia was induced and maintained using inhaled isoflurane.

### CS Preparation

Six young adult male and female C57BL/6 mice were euthanized by CO_2_ asphyxiation and their cecal contents were harvested. The cecal contents were weighed and then homogenized through a 70 µm strainer in 5% dextrose in water to create the CS. CS was then aliquotted and frozen at −80°C until use.

### Polymicrobial Sepsis Model and Survival Studies

The polymicrobial sepsis model was modified as previously described by Wynn et al. (20). Neonatal mice were anesthetized using inhaled isoflurane. They were then gently restrained and CS was injected i.p. at a dose of 0.9 mg/g body weight (BW) for 10-h experiments and 0.175 mg/g BW for survival experiments. Sham mice received an equivalent volume of normal saline i.p. For 10-h experiments, neonates were anesthetized by isoflurane and then euthanized by cardiac puncture. Blood and tissues were collected and preserved for analysis. For survival studies, mice were monitored for a period of 7 days and surviving pups were euthanized by CO_2_ asphyxiation at the end of the study.

### Experimental Reagents

The synthetic α-GalCer analog KRN7000 was purchased from Funakoshi (Tokyo, Japan). KRN was dissolved in dimethyl sulfoxide (DMSO) at a concentration of 1 mg/ml at 80°C. It was then aliquotted and stored at −20°C until use. Prior to injection, the dissolved KRN was diluted in PBS to a final concentration of 25 µg/ml. Neonatal mice were randomized to receive KRN (0.2 µg/g BW) or vehicle (2.5% DMSO in PBS) in the same volume within 1 h after or 30 h prior to CS injection. Lyophilized carrier-free recombinant mouse TGF-β1 was purchased from R&D Systems (Minneapolis, MN, USA). TGF-β1 was reconstituted in 4 mM HCl at a concentration of 50 µg/ml. It was then aliquotted and stored at −20°C until use. Prior to injection the reconstituted TGF-β1 was diluted in PBS to a final concentration of 10 µg/ml. Neonatal mice were randomized to receive TGF-β1 (0.05 µg/g BW) or vehicle (PBS containing 0.8 mM HCl) in the same volume 1 h prior to CS injection.

### Flow Cytometry

Whole livers were harvested from neonatal mice and preserved on ice in RPMI cell culture medium containing 10% FBS, 1% penicillin–streptomycin, 10 mM HEPES, 2 mM l-glutamine, and 50 µM β-mercaptoethanol. Livers were dissociated and homogenized by crushing and filtering through a sterile 70-µm nylon filter. Erythrocytes were then lysed using ammonium-chloride-potassium lysing buffer (Quality Biological, Gaithersburg, MD, USA). Cells were then washed and resuspended in RPMI cell culture medium. The isolated cells were first washed with PBS and stained with Fixability Viability Dye (FVD) eFluor506 (eBioscience, Santa Clara, CA, USA). They were then resuspended in flow cytometry buffer (PBS containing 2% FBS and 0.1% sodium azide) and stained with the following fluorescent antibodies: FITC-CD3, APC/Cy7-CD69 (Biolegend, San Diego, CA, USA) and PE-PBS-57 (α-GalCer)-loaded CD1d tetramer (NIH Tetramer Core Facility, Atlanta, GA, USA). Cells were acquired using a BD LSRFortessa flow cytometer (BD Biosciences, San Jose, CA, USA). Flow cytometry data were analyzed using FlowJo 7.6.5 software (FlowJo LLC, Ashland, OR, USA). On analysis, whole hepatocyte samples were first gated using forward scatter (FSC) and side scatter (SSC) characteristics to identify the lymphocyte population. FSC-H vs. FSC-A gating was then used to exclude doublet cells, and dead cells (FVD eFluor506-positive) were identified and excluded. The FITC-CD3 and PE-α-GalCer-loaded CD1d tetramer double-positive population was identified as iNKT cells. APC/Cy7-CD69 expression on these cells was analyzed compared to an unstained negative control.

### Real-Time Polymerase Chain Reaction (qPCR)

Neonatal mice were euthanized 10 h after sepsis induction by CS injection. Whole lungs were then harvested, flash frozen at −80°C, and crushed over dry ice to a fine powder. Total RNA was extracted from crushed tissue using TRIzol reagent (Invitrogren, Carlsbad). Total RNA (2 µg) underwent reverse transcription using murine leukemia virus reverse transcriptase (Applied Biosystems, Foster City, CA, USA). The qPCR reaction was carried out in a 20-µl final volume containing 0.25 µl each of forward and reverse primers, 2 µl cDNA, 7.5 µl diethyl pyrocarbonate-treated water, and 10 µl Power SYBR Green PCR Master Mix (Applied Biosystems). An Applied Biosystems StepOnePlus real-time PCR machine was used for amplification under the thermal cycling profile of 95°C for 10 min, followed by 40 cycles of 95°C for 15 s and 60°C for 1 min. Mouse β-actin mRNA levels were used for normalization. Relative expression of mRNA was calculated by the 2^−ΔΔCt^ method, and results are expressed as fold change in comparison to the sham group. The sequences for the primers used in this study are as follows: mouse IL-6 5′-CCGGAGAGGAGACTTCACAG-3′ (forward) and 5′-GGAAATTGGGGTAGGAAGGA-3′ (reverse); mouse IL-1β 5′-CAGGATGAGGACATGAGCACC-3′ (forward) and 5′-CTCTGCAGACTCAAACTCCAC-3′ (reverse); mouse keratinocyte chemoattractant (KC) 5′- GCTGGGATTCACCTCAAGAA-3′ (forward) and 5′-ACAGGTGCCATCAGAGCAGT-3′(reverse); mouse macrophage inflammatory protein-2 (MIP-2) 5′-CCCTGGTTCAGAAAATCATCCA-3′ (forward) and 5′-GCTCCTCCTTTCCAGGTCAGT-3′ (reverse); and mouse β-actin 5′-CGTGAAAAGATGACCCAGATCA-3′ (forward) and 5′-TGGTACGACCAGAGGCATACAG-3′ (reverse).

### Measurement of Serum Cytokines

Whole blood samples were allowed to clot for 15–30 min after collection by cardiac puncture. Serum was then separated by centrifugation at 3,000 rpm for 10 min at 4°C and stored at −80°C until use. Serum levels of IL-6, IL-1β, and IFN-γ were measured using enzyme-linked immunosorbent assay kits specific for these cytokines according to manufacturer instructions (BD Biosciences). For the ELISArray, serum samples for each group were pooled (5–10 per group). A multi-analyte ELISArray for the following cytokines was carried out per manufacturer instructions: IL-2, IL-4, IL-5, IL-6, IL-10, IL-12, IL-13, IL-17A, IL-23, IFN-γ, TNF-α, and TGF-β1 (QIAGEN, Germantown, MD, USA). In instances in which serum volumes were insufficient to measure all 12 cytokines, the IFN-γ well was not used and has been omitted from our analysis.

### Histology

Neonatal lung lobes were collected 10 h after CS or sham injection and stored in 10% formalin prior to fixation in paraffin. They were subsequently sectioned into 5-µm cuts and stained with hematoxylin and eosin (H&E). Histologic lung injury was assessed in a blinded fashion using light microscopy evaluation and severity of injury was scored using a system for the assessment of acute lung injury in experimental animals outlined by the American Thoracic Society (21). The weighted score took into account neutrophil infiltration in the alveolar and interstitial spaces, the presence of hyaline membranes, the presence of proteinaceous debris in the airspaces, and the degree of septal thickening. Twenty separate cross sections of the lungs at 400× magnification were assessed for each experimental group, with scores ranging between 0 and 1 for each sample.

### Statistical Analysis

Data are expressed as mean ± SEM and compared by one-way analysis of variance (ANOVA) and the Student–Newman–Keuls test. For survival studies, Kaplan–Meier curves are presented and groups were compared using the log-rank test. Differences in values were considered significant if *p* < 0.05.

## Results

### Timing of KRN Treatment Affects Systemic Inflammation in Neonatal Sepsis

**Figure 1:**
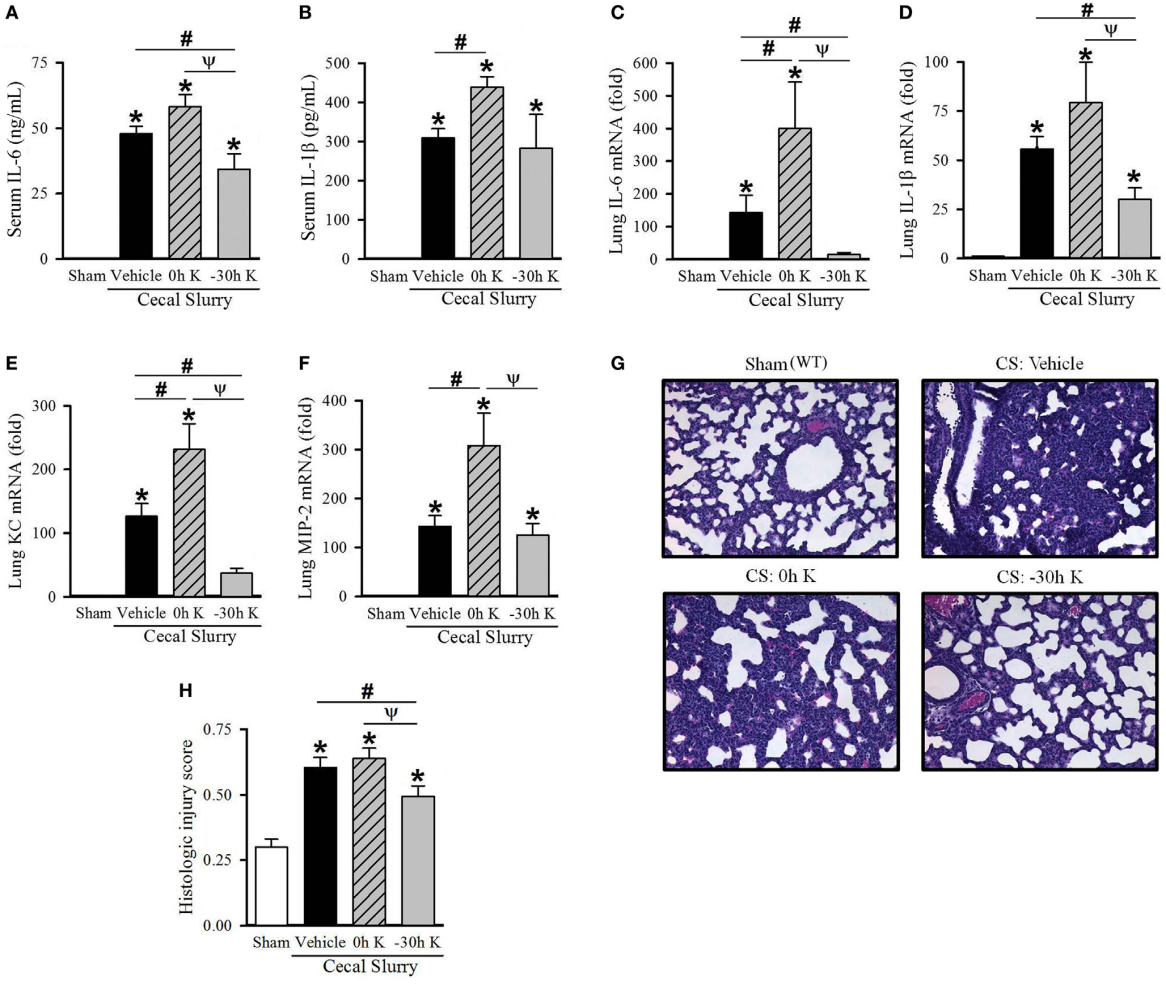
Effect of KRN treatment timing on inflammation and pulmonary injury in septic neonatal mice. Neonatal C57BL/6 pups received KRN (0.2 µg/g BW) or vehicle (2.5% dimethyl sulfoxide in PBS) intraperitoneal (i.p.) within 1 h after (0 h K) or 30 h prior to (−30 h K) sepsis induction. Sepsis was induced by i.p. injection of cecal slurry (0.9 mg/g BW); serum and lungs were harvested 10 h later. Serum levels of **(A)** IL-6 and **(B)** IL-1β were measured by enzyme-linked immunosorbent assay. The mRNA levels of **(C)** IL-6, **(D)** IL-1β, **(E)** KC, and **(F)** MIP-2 in the lungs were determined by qPCR. **(G)** Shown are representative hematoxylin and eosin-stained sections of the lung from sham, vehicle-treated, 0 h KRN-treated and −30 h KRN-treated animals at 200× magnification. **(H)** Histologic lung injury score was calculated for each group, with a maximum possible score of 1. Data expressed as mean ± SEM (*n* = 6–8 per group) and compared by one-way analysis of variance (*p < 0.05 vs. Sham, ^#^*p* < 0.05 vs. Vehicle, ^Ψ^*p* < 0.05 vs. 0 h K).

We first determined the effect of KRN treatment within 1 h after (0 h K) or 30 h prior to (−30 h K) CS injection on inflammation in the neonates. We used the 30-h time point for pretreatment as the earliest possible when considering technical feasibility and mothers’ behavior (22). Ten hours after CS injection blood was collected and serum cytokine levels were measured. Vehicle-treated septic neonates demonstrated a significant increase in serum levels of the proinflammatory cytokines IL-6 (48 ± 3 ng/ml) and IL-1β (310 ± 23 pg/ml) compared to sham pups (undetectable, Figures 1A,B). Treatment with KRN shortly after sepsis induction resulted in a worsening of systemic inflammation, with a further elevation in IL-6 levels (58 ± 5 ng/ml) and a significant increase in IL-1β levels (439 ± 27 pg/ml, Figures 1A,B). Thirty-hour KRN pretreatment, on the other hand, significantly reduced serum levels of IL-6 compared to vehicle- and 0 h KRN-treated septic neonatal mice (34 ± 6 ng/ml, Figure 1A). However, not much change in IL-1β levels with KRN pretreatment compared to the vehicle (Figure 1B).

### Timing of KRN Treatment Affects Pulmonary Inflammation and Injury in Neonatal Sepsis

Lung injury is an important contributor to morbidity and mortality in neonatal sepsis (23, 24). We next examined the effect of KRN treatment timing on pulmonary inflammation in our polymicrobial sepsis model. Ten hours after sepsis induction, expression of IL-6 and IL-1β as well as the neutrophil chemoattractants KC and MIP-2 in the lungs of neonatal mice were measured. Vehicle-treated septic neonates demonstrated a significant increase in IL-6, IL-1β, KC, and MIP-2 expression compared to sham (144-, 56-, 127-, and 143-fold, respectively; Figures 1C–F). Furthermore, 0 h KRN treatment exacerbated this inflammation with significant increases in IL-6, KC, and MIP-2 expression compared to the vehicle-treated pups (400-, 231-, and 308-fold of sham, respectively; Figures 1C,E,F). In contrast, −30 h KRN treatment attenuated inflammation in the lungs as it had systemically, with significant decreases in expression of IL-6, IL-1β, and KC compared to vehicle-treated septic mice (15-, 30-, and 37-fold of sham, respectively; Figures 1C–E).

We next sought to correlate the differential effect of KRN treatment timing on systemic and pulmonary inflammation with lung injury at the histologic level. Representative sections of the lungs from each group are shown in Figure 1G. Sham pups had a mean histologic injury score of 0.30 ± 0.03 (Figure 1H), based on the American Thoracic Society assessment with the maximum score of one. This was significantly increased in vehicle-treated pups to 0.61 ± 0.04 (Figure 1H). Pups treated with KRN after sepsis induction demonstrated a similar level of histologic lung injury to vehicle-treated pups (0.64 ± 0.04, Figure 1H). Consistent with our findings of decreased systemic levels of proinflammatory cytokines and decreased cytokine and chemokine expression in the lungs, −30 h KRN-treated pups demonstrated an attenuation of histologic lung injury compared to vehicle- and 0 h KRN-treated septic neonates (0.49 ± 0.04, Figure 1H).

### Kinetic Analysis of iNKT Cell Activation in Neonatal Mice

**Figure 2:**
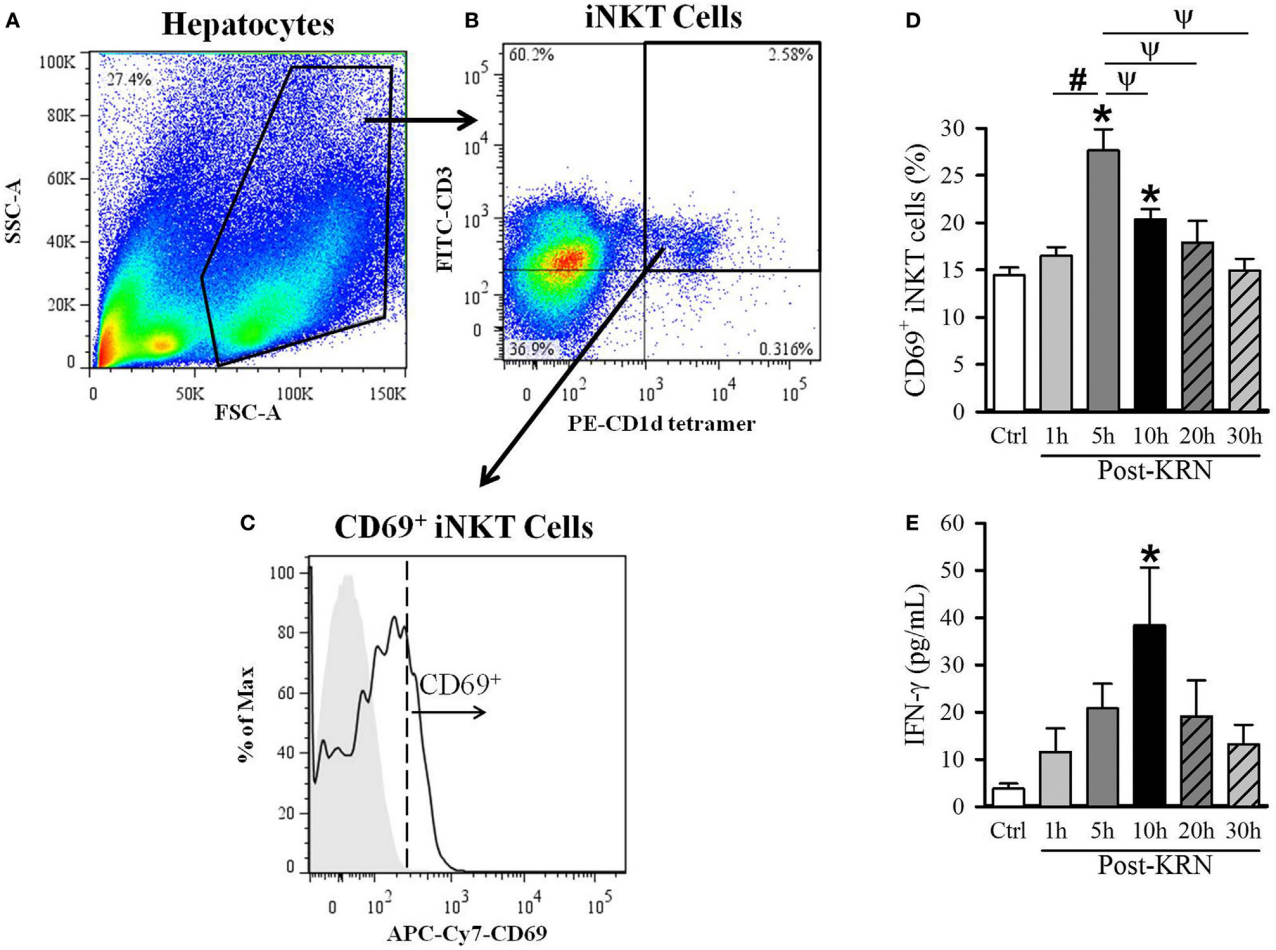
Kinetic analysis of KRN treatment on hepatic invariant natural killer T (iNKT) cell activation in neonatal mice. Serum and liver from C57BL/6 pups received KRN (0.2 µg/g BW) intraperitoneal were harvested at 1, 5, 10, 20, and 30 h after KRN administration or sham. Liver was dissociated through a 70-µm strainer to isolate hepatocytes, followed by staining and flow cytometry analysis. A representative sample showing flow cytometry gating strategy is shown. **(A)** Total cells isolated from the liver were gated for the live lymphocyte population based on forward scatter (FSC) and side scatter (SSC) characteristics. **(B)** The iNKT cell population was then identified by gating for the FITC-CD3 and PE-CD1d tetramer double-positive cell population. **(C)** Flow cytometric analysis of surface CD69 expression on the gated hepatic iNKT cells. Shaded area indicates negative control. **(D)** Flow cytometric analysis of surface CD69 expression on gated hepatic iNKT cells at each time point. **(E)** Interferon (IFN)-γ levels in the serum were measured by enzyme-linked immunosorbent assay at each time point. Data expressed as mean ± SEM (*n* = 6–16 per group) and compared by one-way analysis of variance (*p < 0.05 vs. Ctrl, ^#^*p* < 0.05 vs. 1 h, ^ψ^*p* < 0.05 vs. 5 h).

Given the differential effects of KRN treatment at different treatment time points in neonatal sepsis, we next determined the activation kinetics of neonatal iNKT cells after KRN administration. It has been reported that iNKT cells are abundantly found in the liver of adult mice (13, 25, 26). As such, we examined the activation status of hepatic iNKT cells at 1, 5, 10, 20, and 30 h in healthy neonatal mice after KRN treatment by measuring CD69 expression using flow cytometry. The total hepatic lymphocyte population was gated by FSC and SSC characteristics (Figure 2A). We further excluded doublets and dead cells as described in the Section “Materials and Methods.” We then used FITC-labeled CD3 antibodies and PE-labeled α-GalCer-loaded CD1d tetramer antibodies to identify iNKT cells (Figure 2B). We then looked at the level of activation of this subset using APC/Cy7-labeled CD69 antibodies (Figure 2C).

The frequency of CD69-positive iNKT cells in the liver incre-ased from 14.4 ± 0.8% in untreated controls to a peak of 27.6 ± 2.2% 5 h after KRN administration (Figure 2D). We also measured serum levels of IFN-γ, which is released by activated iNKT cells (27). Serum level of IFN-γ in control pups was 3.8 ± 1.1 pg/ml and reached a peak of 38.4 ± 12.2 pg/ml at 10 h after KRN administration (Figure 2D). Both CD69 expression and IFN-γ serum levels returned to near baseline levels at 30 h (15.0 ± 1.2% and 13.2 ± 4.2 pg/ml, respectively, Figures 2D,E). These data reveal the activated status of iNKT cells in the neonates with KRN treatment before they were subjected to sepsis induction.

### Timing of KRN Treatment Affects iNKT Cell Activation Status in Neonatal Sepsis

**Figure 3:**
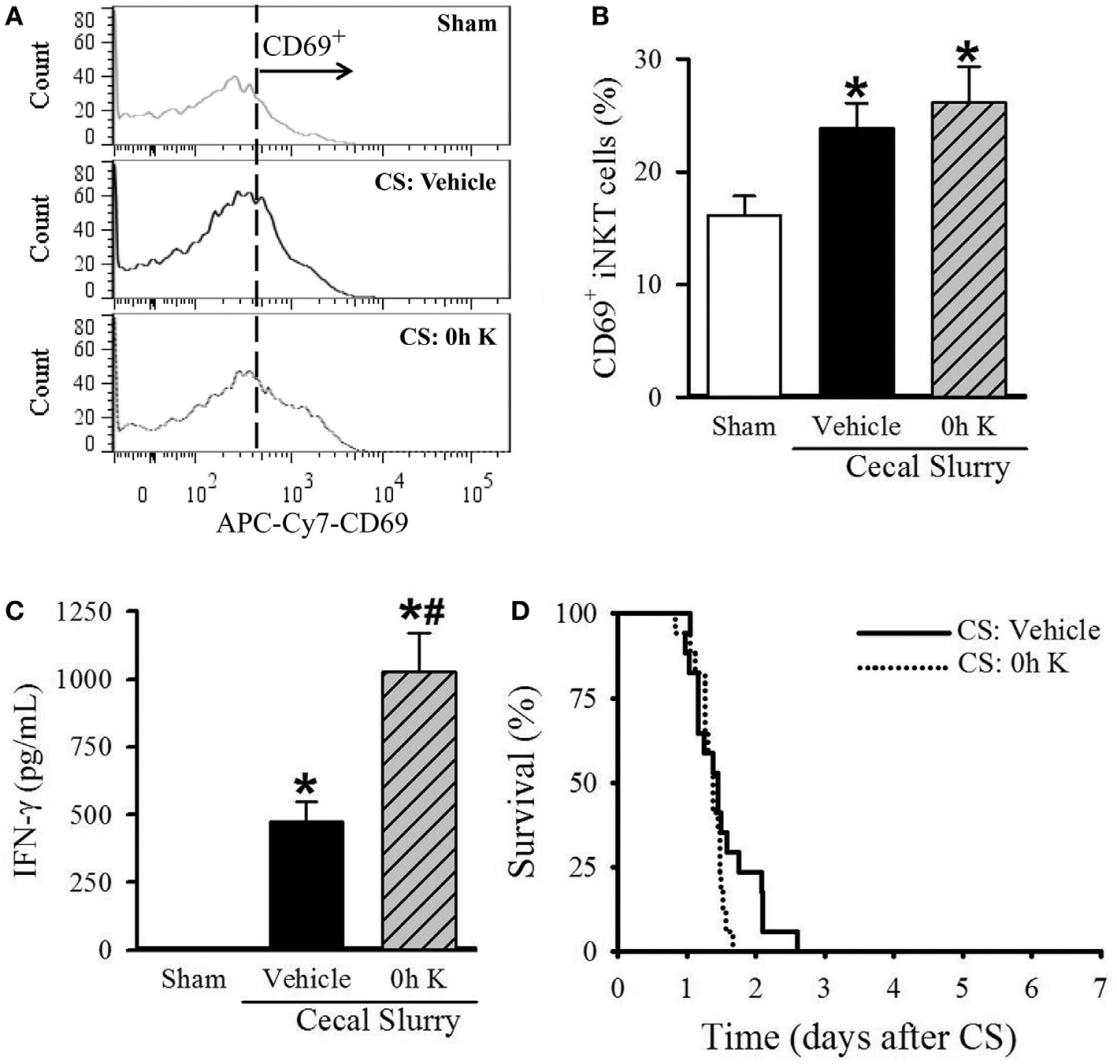
Effect of 0 h KRN treatment on invariant natural killer T (iNKT) cell activation and survival in septic neonatal mice. Sepsis was induced in neonatal C57BL/6 pups by intraperitoneal (i.p.) injection of cecal slurry (CS, 0.9 mg/g BW). Pups then received KRN (0.2 µg/g BW) or vehicle (2.5% dimethyl sulfoxide in PBS) i.p. within 1 h. Serum and liver were harvested 10 h later. **(A)** Representative histogram of CD69 expression on hepatic iNKT cells. **(B)** Flow cytometric analysis of surface CD69 expression on gated hepatic iNKT cells. **(C)** Interferon (IFN)-γ levels in the serum were measured by enzyme-linked immunosorbent assay. Data expressed as mean ± SEM (*n* = 6–9 per group) and compared by one-way analysis of variance (*p < 0.05 vs. Sham, ^#^*p* < 0.05 vs. Vehicle). **(D)** Another set of pups received the same dose of KRN or vehicle within 1 h after sepsis induction by CS (0.175 mg/g BW) and survival was monitored for seven days (*n* = 17 per group, *p* = 0.129, log-rank test).

After determining the kinetic response of iNKT cells to KRN administration, we next examined the effect of KRN treatment timing during sepsis on iNKT cells. We isolated the cells from the livers of sham and septic pups with vehicle and 0 h KRN treatments at 10 h after CS injection and analyzed them by flow cytometry. Vehicle-treated septic pups demonstrated a significant increase in the frequency of CD69 expression on hepatic iNKT cells compared to sham pups (23.9 ± 2.3 vs. 16.1 ± 1.7%), and this was further increased in 0 h KRN-treated pups (26.1 ± 3.2%, Figures 3A,B). In addition, vehicle-treated pups had a significant increase in serum levels of IFN-γ 10 h after CS injection compared to sham pups (1.3 ± 0.7 vs. 474.7 ± 73.6 pg/ml), which was further increased in the 0 h KRN-treated pups (1,027.8 ± 144.0 pg/ml, Figure 3C). In a 7-day survival study, the 0 h KRN-treated septic pups died earlier than the vehicle-treated group (Figure 3D).

**Figure 4:**
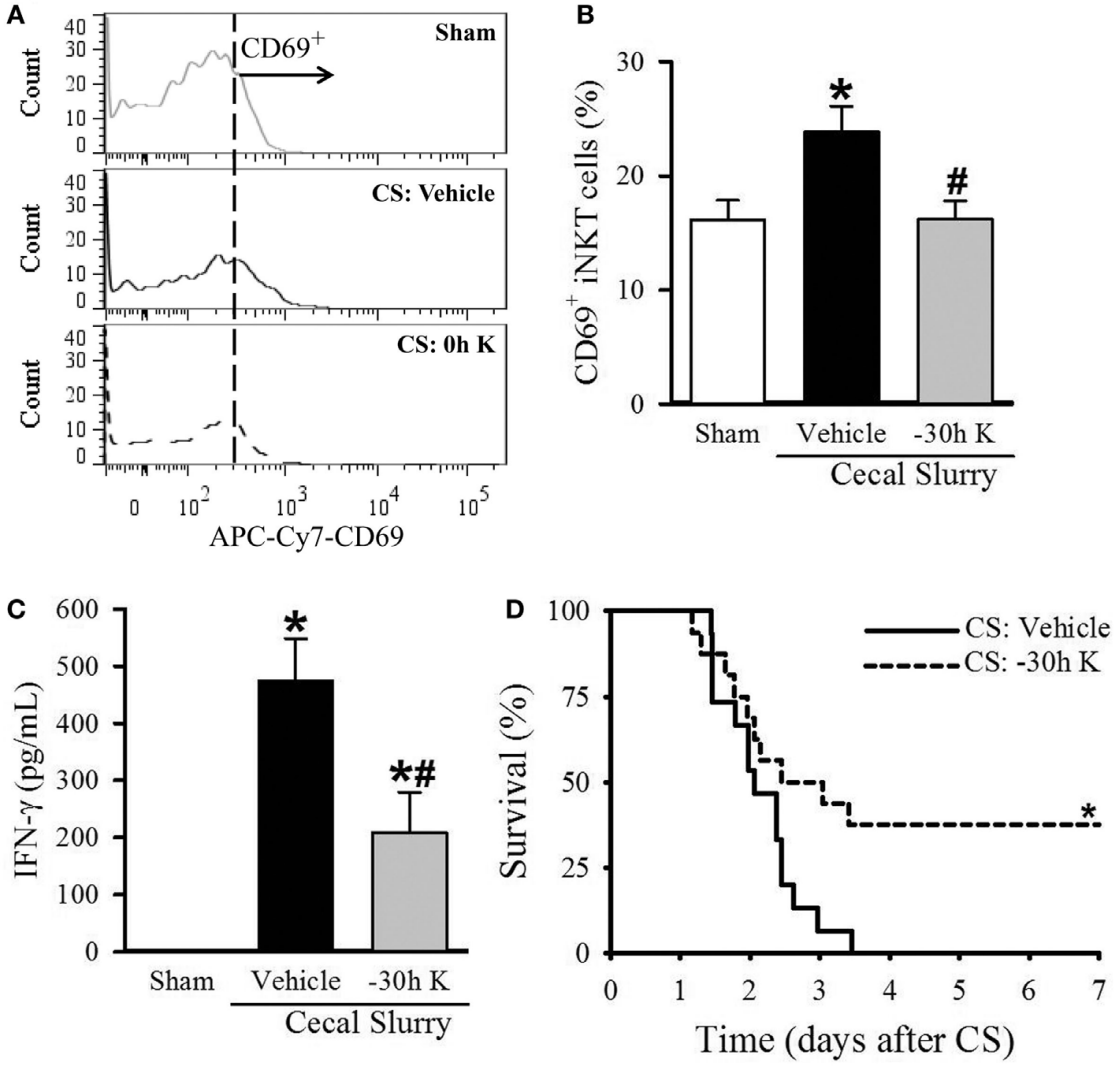
Effect of −30 h KRN treatment on invariant natural killer T (iNKT) activation cell activation and survival in septic neonatal mice. Neonatal C57BL/6 pups received KRN (0.2 µg/g BW) or vehicle (2.5% dimethyl sulfoxide in PBS) intraperitoneal (i.p.) 30 h prior to (−30 h K) sepsis induction. Sepsis was then induced by i.p. injection of cecal slurry (CS, 0.9 mg/g BW). Serum and liver were harvested 10 h later. **(A)** Representative histogram of CD69 expression on hepatic iNKT cells. **(B)** Flow cytometric analysis of surface CD69 expression on gated hepatic iNKT cells. **(C)** Interferon (IFN)-γ levels in the serum were measured by enzyme-linked immunosorbent assay. Data expressed as mean ± SEM (*n* = 5–9 per group) and compared by one-way analysis of variance (*p < 0.05 vs. Sham, ^#^*p* < 0.05 vs. Vehicle). **(D)** Another set of pups received the same dose of KRN or vehicle 30 h prior to sepsis induction by CS (0.175 mg/g) and survival was monitored for 7 days (*n* = 15–16 per group, *p = 0.026 vs. Vehicle, log-rank test).

Conversely, septic pups that received −30 h KRN treatment demonstrated a decrease in CD69 expression on hepatic iNKT cells compared to vehicle-treated pups (16.2 ± 1.6 vs. 23.9 ± 2.2%, Figures 4A,B), as well as a decrease in systemic levels of IFN-γ (209.1 ± 70.3 vs. 474.7 ± 73.6 pg/ml, Figure 4C). This reduction in iNKT cell activation corresponds to the previously mentioned reduction in systemic and pulmonary inflammation and histologic lung injury. In addition, −30 h KRN treatment resulted in a remarkable 38% survival rate at 7 days compared to no survival in the vehicle-treated group (*p* = 0.026, log-rank test; Figure 4D).

### The Protective Effect of KRN Pretreatment Is Dependent on CD1d

**Figure 5:**
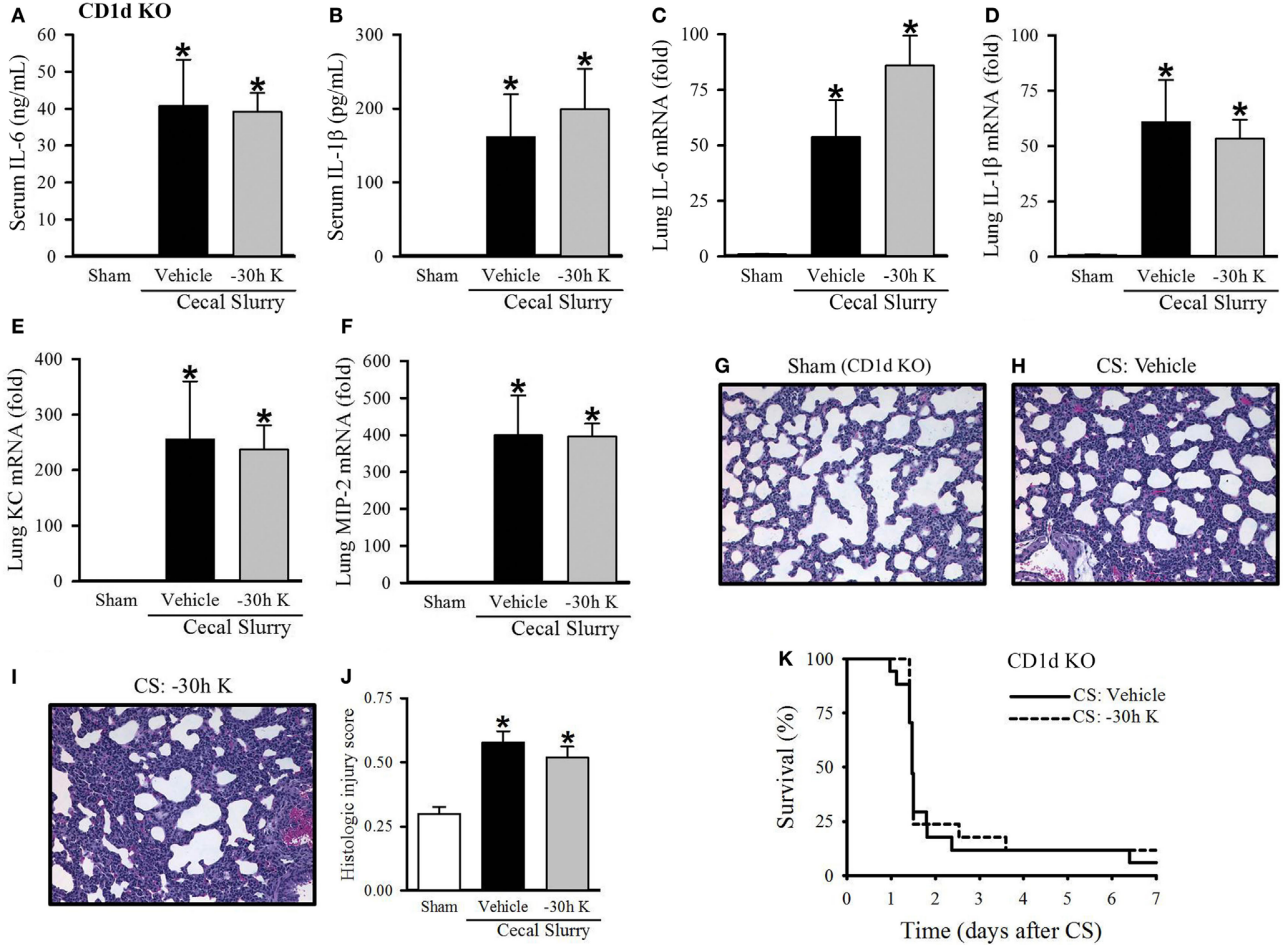
Effect of −30 h KRN treatment on inflammation, pulmonary injury, and survival in septic CD1d knockout (KO) neonatal mice. Neonatal CD1d KO pups received KRN (0.2 µg/g BW) or vehicle (2.5% dimethyl sulfoxide in PBS) intraperitoneal (i.p.) 30 h prior to sepsis induction. Sepsis was induced by i.p. injection of cecal slurry (CS) (0.9 mg/g BW); serum and lungs were harvested 10 h later. Serum levels of **(A)** IL-6 and **(B)** IL-1β were measured by enzyme-linked immunosorbent assay. The mRNA levels of **(C)** IL-6, **(D)** IL-1β, **(E)** keratinocyte chemoattractant, and **(F)** macrophage inflammatory protein-2 in the lungs were determined by qPCR. **(G–I)** Shown are representative hematoxylin and eosin-stained sections of the lung from **(G)** sham, **(H)** vehicle-, and **(I)** −30 h KRN-treated mice at 200× magnification. **(J)** Histologic lung injury score was calculated for each group with a maximum possible score of 1. Data expressed as mean ± SEM (*n* = 6–8 per group) and compared by one-way analysis of variance (*p < 0.05 vs. Sham). **(K)** Another set of pups received the same dose of KRN or vehicle 30 h prior to sepsis induction by CS (0.175 mg/g) and survival was monitored for 7 days (*n* = 17 per group, *p* = 0.667, log-rank test).

We next wished to confirm that the protective effect seen with 30 h KRN pretreatment was mediated through CD1d. First, we looked at the effect of −30 h KRN treatment on systemic inflammation in septic CD1d KO neonatal mice. At 10 h after sepsis induction, there was no difference in levels of the proinflammatory cytokines IL-6 or IL-1β between vehicle- and KRN-pretreated pups (41 ± 13 vs. 39 ± 5 ng/ml and 162 ± 58 vs. 199 ± 55 pg/ml, respectively; Figures 5A,B). On qPCR analysis, there was no statistical difference in expression of IL-6 or IL-1β in the lungs of vehicle- and KRN-pretreated septic CD1d KO pups (54- vs. 86-fold and 61- vs. 53-fold of sham, respectively; Figures 5C,D), nor was there a difference in expression of the neutrophil chemoattractants KC and MIP-2 between the two groups (255- vs. 238-fold and 399- vs. 396-fold of sham, respectively; Figures 5E,F).

CD1d KO sham pups had histologic lung injury scores of 0.30 ± 0.03, similar to C57BL/6 sham pups (Figure 5G). Both vehicle- and −30 h KRN-treated septic CD1d KO neonates demonstrated increased lung injury compared to sham pups, although there was no statistical difference in injury score between the two septic groups (0.58 ± 0.04 and 0.52 ± 0.04, respectively; Figures 5G–J). There was also no difference in 7-day survival between KRN-pretreated and vehicle-treated CD1d KO pups (Figure 5K).

### Kinetic Analysis of KRN Treatment on Serum Cytokine Levels in Neonatal Mice

Activated iNKT cells release several cytokines and affect many other types of immune cells (27). We next examined whether a change in cytokine expression profile is associated with the protective effect of 30 h KRN pretreatment on inflammation, lung injury, and survival in neonatal sepsis. We utilized a multi-analyte ELISArray to measure the relative levels of various cytokines in the serum of neonatal mice at 1, 5, 10, 20, and 30 h after KRN treatment. Due to the limited volume of blood that could be drawn from each neonate, we pooled samples from neonates at each time point (5–10 samples per group) to perform the cytokine array analysis. As shown in Figure S1 in Supplementary Material, most notably, there was a slight increase in IL-2, a cytokine implicated in lymphocyte proliferation and type I immunity (Th1) at 5 h after KRN administration. This corresponds to the time point at which peak frequency of CD69 expression on iNKT cells in the neonatal liver was seen. Then, at the protective 30-h time point, the absorbance value for TGF-β1, considered Th2- or Th17-associated depending on the cytokine milieu, reached three times that of the control sample. Additional Th1- (IL-6, IL-12, TNF-α), Th2- (IL-4, IL-5, IL-10, IL-13), and Th17- (IL-17A, IL-23) associated cytokines were also analyzed. In addition to TGF-β1, but to a lesser degree, IL-4 was increased at 30 h relative to control levels. TNF-α, IL-6, IL-10, IL-17A, and IL-23 showed smaller increases at 30 h relative to control, while IL-2, IL-12, IL-5, and IL-13 were near baseline levels.

### Effect of TGF-β1 Administration on Inflammation in Neonatal Sepsis

**Figure 6:**
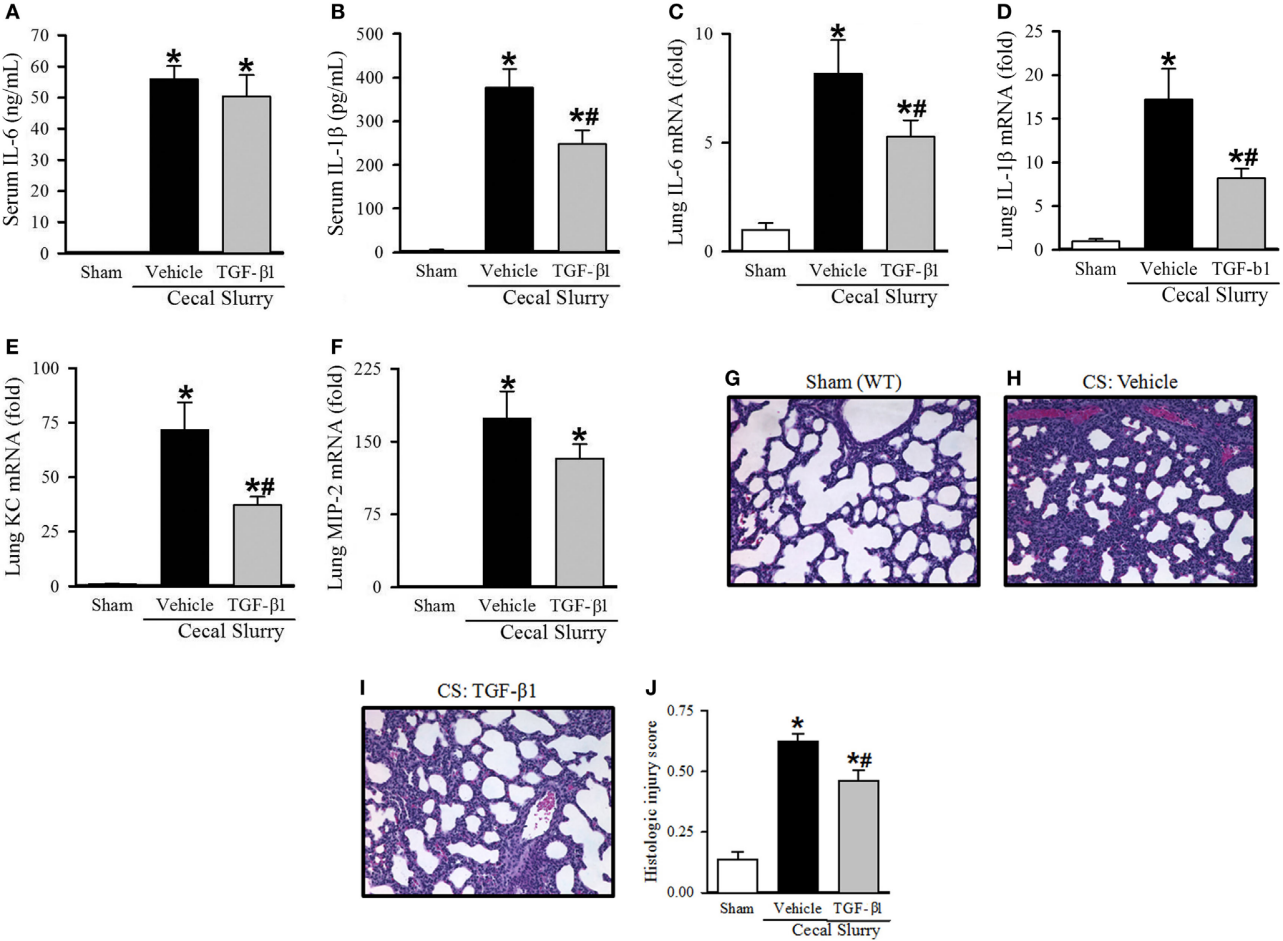
Effect of transforming growth factor (TGF)-β1 administration inflammation and pulmonary injury in septic neonatal mice. Neonatal C57BL/6 pups received recombinant mouse TGF-β1 (0.05 µg/g BW) or vehicle (PBS containing 0.8 mM HCl) intraperitoneal (i.p.) 1 h prior to sepsis induction. Sepsis was induced by i.p. injection of cecal slurry (0.9 mg/g BW); serum and lungs were harvested 10 h later. Serum levels of **(A)** IL-6 and **(B)** IL-1β were measured by enzyme-linked immunosorbent assay. The mRNA levels of **(C)** IL-6, **(D)** IL-1β, **(E)** keratinocyte chemoattractant, and **(F)** macrophage inflammatory protein-2 in the lungs were determined by qPCR. **(G–I)** Shown are representative hematoxylin and eosin-stained sections of the lung from **(G)** sham, **(H)** vehicle-, and **(I)** TGF-β1-treated mice at 200× magnification. **(J)** Histologic lung injury score was calculated for each group with a maximum possible score of 1. Data expressed as mean ± SEM (*n* = 7–8 per group) and compared by one-way analysis of variance (*p < 0.05 vs. Sham, ^#^*p* < 0.05 vs. Vehicle).

After identifying a marked increase in serum TGF-β1 at 30 h after KRN treatment, we further determined whether it played a role in protecting neonates against sepsis. Neonatal mice received TGF-β1 (0.05 µg/g BW) or vehicle (PBS containing 0.8 mM HCl) i.p. within 1 h prior to CS injection. Ten hours after sepsis induction, vehicle-treated pups demonstrated a significant increase in serum levels of IL-6 and IL-1β compared to sham pups (55.9 ± 4.2 vs. 0.1 ± 0.1 ng/ml and 377.2 ± 42.6 vs. 4.6 ± 2.6 pg/ml, respectively; Figures 6A,B). TGF-β1 treatment did not significantly attenuate systemic levels of IL-6 (50.4 ± 6.9 ng/ml, Figure 6A), but did result in a significant decrease in systemic levels of IL-1β compared to vehicle treatment (248.1 ± 32.0 pg/ml, Figure 6B).

Turning our attention to pulmonary inflammation, on qPCR analysis there was an eight-fold increase in IL-6 expression and a 17-fold increase in IL-1β expression in vehicle-treated septic pups compared to sham pups (Figures 6C,D). TGF-β1 significantly decreased IL-6 and IL-1β expression in the lungs to just five- and eight-fold of sham, respectively (Figures 6C,D). KC and MIP-2 expression were also increased in the lungs of vehicle-treated septic pups compared to sham (72- and 173-fold, respectively), and were attenuated in the TGF-β1-treated group (37- and 132-fold, respectively, Figures 6E,F).

### Effect of TGF-β1 Administration on Lung Injury in Neonatal Sepsis

Given the clear reduction in systemic and pulmonary inflammation seen with TGF-β1 treatment in septic neonates, we then examined the effect of this treatment on lung injury. Representative H&E-stained sections from sham, vehicle- and TGF-β1-treated pups are show in Figures 6G–I. Sham pups demonstrated a mean histologic injury score of 0.14 ± 0.03 (Figure 6J). This score was significantly increased to 0.63 ± 0.03 in vehicle-treated septic pups, while TGF-β1-treated pups had significantly attenuated histologic lung injury with a mean score of 0.46 ± 0.04 (Figure 6J).

### Effect of KRN Treatment on Serum Cytokine Levels in CD1d KO Neonatal Mice

To further validate whether elevation of TGF-β1 after KRN treatment was mediated by CD1d, we performed the multi-analyte ELISArray in the serum of control and KRN-treated CD1d KO neonatal mice. Contrary to the three-fold increase seen in wild-type mice, there was no change in relative levels of TGF-β1 in CD1d KO mice at 30 h after KRN treatment (Figure S2 in Supplementary Material). In addition, the change in several cytokines in the wild-type neonatal mice was diminished in the CD1d KO neonatal mice following KRN stimulation. This finding further supports that activation of iNKT cells by KRN is mediated by CD1d.

## Discussion

In this study, we have demonstrated that treatment of neonatal mice with the α-GalCer analog KRN7000 at the time of sepsis induction by CS results in worsening of systemic and pulmonary inflammation and histologic lung injury and accelerates mortality. In contrast, 30-h KRN pretreatment reduces systemic and pulmonary inflammation, decreases histologic lung injury, and improves survival in neonatal sepsis. We have further conducted a 30-h kinetic analysis of neonatal iNKT cell activation and serum levels of several cytokines after KRN treatment. We have demonstrated that KRN’s protective effect is mediated by CD1d. We have identified an increase in serum levels of TGF-β1 in neonates at 30 h after KRN stimulation. Finally, we have demonstrated that administration of TGF-β1 attenuates systemic and pulmonary inflammation and lung injury in neonatal mice, establishing a mechanism through which KRN pretreatment exerts its protective effect.

We have shown that the timing of iNKT cell activation by KRN critically affects outcomes in a neonatal model of polymicrobial sepsis. When given at the time of sepsis induction by CS, KRN worsened injury. This is in line with a CS sepsis model in adult mice, in which KRN treatment did not improve survival relative to vehicle-treated controls when given within 20 min of CS injection (28). However, in an endotoxemia model in adult mice, KRN treatment increased survival, reduced serum levels of AST and ALT, and protected mice from BW loss when administered within 2 h before or after lipopolysaccharide (LPS) challenge (29). Of note, these mice were pre-challenged with low dose LPS 24 h before the second LPS administration. Such discrepancy in outcomes suggests that the effect of KRN treatment is dependent on the type of infection. Another potential factor that may contribute to varying outcomes between 0 h and −30 h treatment groups is the developmental stage of iNKT cells. Recognition of CD1d-loaded endogenous lipid antigen(s) on CD4/CD8-double-positive (DP) thymocytes with interactions between signaling lymphocyte activation molecule (SLAM) family members is essential for the development of iNKT cells. After differentiating into different iNKT cell subsets in the thymus, these cells then distribute in peripheral organs (30). The role of SLAM signaling on changes in cytokine levels at different time points after KRN treatment needs further investigation. Also, it will be interesting to look at differences in treatment timing over age ranges of hours rather than days to further elucidate the effect of immune system maturation on iNKT cell activation in the future.

In contrast to what was seen in the KRN posttreatment experiment, KRN pretreatment 30 h before CS injection resulted in a reduction in systemic and pulmonary inflammation and histologic lung injury. Furthermore, we demonstrated a significant increase in 7-day survival to 38 from 0% survival in the vehicle-treated group. Likewise, adult mice that received KRN pretreatment 6 to 12 days before LPS injection also show reduced organ injury and improved survival (31). However, in our neonatal model, 30-h KRN pretreatment was sufficient to show the protective effect, while pretreatment at least 72 h prior to LPS injection was required to see a protective effect in adult mice. Due to the age of the neonates used in our study, we cannot extend the time of KRN pretreatment further to test whether earlier KRN treatment will provide better protection. It has been reported that administration of KRN to pregnant mice induces abortion through decidual iNKT cell activation (32). To our knowledge there has not been a study of KRN pretreatment in a polymicrobial sepsis model such as CS or cecal ligation and puncture in adult animals; such study is warranted in order to more directly compare the response of neonatal and adult iNKT cells to KRN pretreatment in sepsis. Nevertheless, the benefits demonstrated with KRN pretreatment in the neonate highlight the potential of modulating the neonatal immune system, including *via* iNKT cell activation, for prophylactic benefit in populations particularly at risk for sepsis (i.e., premature and very low birth weight infants). Moreover, with the observation of opposite effects between pre- and immediate posttreatment with KRN, it will be worthwhile to explore the effects of delayed treatment (i.e., more than 1 h after CS injection) at multiple time points following sepsis induction in neonates in future studies.

To understand the effect of KRN treatment on iNKT cells, we monitored the activation status of iNKT cells in neonatal mice for 30 h after treatment. We focused on the hepatic subset of iNKT cells because of its abundance relative to other compartments. It is estimated that NKT cells compromise 30% of the lymphoid population in the mouse liver, and up to 50% of the hepatic lymphoid population in humans (25, 33). On the other hand, iNKT cells make up only 1–2% of lymphocytes in the mouse spleen and an estimated 0.1–0.2% of peripheral blood T cells in humans (13). We found that frequency of the activation marker CD69 expression in these hepatic iNKT cells increased from a baseline level of 14% to a peak of 28% at 5 h after KRN administration. Frequency of CD69 expression then decreased to baseline levels at 30 h after KRN administration. These findings correlated with serum levels of IFN-γ, a cytokine secreted by iNKT cells as well as several downstream effector cells after iNKT activation, which increased modestly after KRN administration to a maximum of 38 pg/ml at 10 h. We also measured IL-4 in the serum, but saw no significant increase in levels compared to the control at any time point (data not shown).

The kinetics of iNKT cell activation in neonates that we demon-strated here are different from those that have been reported in adult mice. In response to KRN injection in adult mice, NKT cells become activated within minutes to hours and express activation markers including CD69 (27). Within 1 h of KRN treatment IL-4 is produced by NKT cells, and this is followed by an increase in IFN-γ production, which peaks at 24 h after injection (27, 34). Given that we see a serum increase in IFN-γ and iNKT cell activation at five to 10 h after KRN administration in the neonates, contrary to the peak at 24 h in adult mice. In addition, serum IL-4 levels in the neonates did not change during any of the time points in our study. There may be an early increase in IL-4 production in neonatal iNKT cells that is not captured in our studies within 1 h.

The changes in iNKT cell activation and serum IFN-γ levels 5–10 h after KRN administration in healthy neonates correspond to the exacerbated inflammatory response seen with 0 h KRN treatment in septic neonates. In our sepsis model, vehicle-treated animals demonstrated a significant increase in both frequency of CD69 expression on iNKT cells and serum IFN-γ levels at 10 h after CS injection. With 0 h KRN treatment, there was an increase in frequency of CD69 expression on iNKT cells and a significant increase in serum IFN-γ levels compared to the vehicle. IFN-γ plays a critical role in initiating and sustaining the innate immune response to infection. Its production by natural killer cells is important for controlling infection in the early stages of the response, before the adaptive immune system has been activated (35). In addition, CD4^+^ T cells exposed to IFN-γ in the early phase of infection tend to differentiate into Th1 type effector cells, rather than Th2 cells, leading to a proinflammatory state (35). Given that in our kinetic analysis we saw increases in both frequency of CD69 expression and IFN-γ levels at five to 10 h after KRN administration, and our understanding of IFN-γ as a proinflammatory cytokine, this synergistically detrimental effect at 10 h following sepsis induction and KRN administration is not surprising.

In our kinetic analysis, CD69 expression had returned to baseline by the 30-h time point, as had IFN-γ levels in the serum in neonates. Therefore, at the time of CS injection in the 30 h KRN-pretreated neonates, their iNKT cells were not in the activated state. However, we still observed a relative and significant decrease in both frequency of CD69 expression on iNKT cells and in serum IFN-γ levels in the 30 h KRN-pretreated neonates, compared to the vehicle. It is possible that KRN-stimulated iNKT cells develop tolerance that blunts the secondary insult by CS injection. In addition, activated iNKT cells may stimulate other immune cells to protect against the insult of CS injection 30 h later. In our multi-analyte ELISArray study, we observed a change in several cytokines in the serum of neonates 30 h after KRN stimulation, which further supports the notion that other immune cells are activated after iNKT cell stimulation.

KRN is a synthetic α-GalCer analog and CD1d ligand. We next determined whether KRN was acting through this pathway rather than an alternative one to affect outcomes in neonatal sepsis. In order to study this, we utilized CD1d KO mice. These mice harbor deletions of the *CD1d1* and *CD1d2* loci, which prohibit the normal development of NKT cells. KRN was administered to CD1d KO neonates 30 h prior to sepsis induction by CS, as had been done in the C57BL/6 wild-type neonates. Remarkably, 10 h after sepsis induction, there were no differences in serum levels of IL-6 or IL-1β, or in fold induction of IL-6, IL-1β, KC, or MIP-2 mRNA expression in the lungs between vehicle- and KRN-pretreated CD1d KO pups, in contrast to what was seen in the wild-type neonates. In addition, while sepsis induced histologic lung injury in these pups compared to the sham, there was not a protective effect in the KRN-pretreated CD1d KO pups. The protective effect of KRN pretreatment was abrogated in the KO animals, confirming our suspicion that CD1d mediates KRN’s effect. This is consistent with previous findings in adult animals that KRN administration *in vivo* resulting in the activation of NKT cells is lost in CD1d-deficient mice (36). To identify potential cytokines mediating this protective effect, we next used a multi-analyte ELISArray to assess relative cytokine levels in the serum at various time points for 30 h after KRN administration. At 5 h, there was a modest but noticeable increase in the Th1-associated proinflammatory cytokine IL-2. This is consistent with our initial quantitative analysis of serum levels of IFN-γ, another Th1-associated cytokine, which showed a peak at 10 h after KRN administration, as well as our flow cytometric analysis of hepatic iNKT cells, which demonstrated increased frequency of CD69 expression at 5 h after KRN administration. Whether an overall Th1 phenotype in the immune response at five to 10 h after KRN administration might contribute to the exacerbated inflammation and accelerated mortality seen in the 0 h KRN treatment group is an interesting possibility that warrants further investigation.

At our protective 30-h time point, there was a marked increase in serum TGF-β1 levels. TGF-β1 has been shown to regulate multiple immune cell types. It inhibits aberrant T cell expansion, inhibits myeloid proliferation, downregulates production of reactive oxygen species in myeloid cells, acts as a chemotactic factor for monocytes, and negatively regulates the proinflammatory NF-κB pathway (37). Considering the diverse protective effects of TGF-β and our finding of elevated serum levels of TGF-β1 in neonatal mice 30 h after KRN administration, we then determined the effect of TGF-β1on septic neonates. In fact, we found that recombinant murine TGF-β1 administered to C57BL/6 neonates 1 h prior to sepsis induction by CS decreased systemic and pulmonary inflammation and decreased pulmonary injury compared to vehicle. The beneficial effect of TGF-β1 administration has also been demonstrated in a neonatal rat model of necrotizing enterocolitis (NEC), in which it reduced NEC incidence and systemic inflammation in addition to protecting the integrity of the gut epithelium (38). Similarly, in adult rats with endotoxemia, TGF-β1 reduced inflammation and improved survival (39).

To further confirm that TGF-β1 mediates the protective effect of 30-h KRN pretreatment, we determined serum cytokine levels in CD1d KO neonates at 30 h after KRN administration. Compared to wild-type neonates, changes in most cytokine levels, especially TGF-β1, were diminished in the KRN-stimulated CD1d KO neonates. As we have demonstrated, the protective effect of 30 h KRN pretreatment is lost in the CD1d KO neonates. Taken together, these results indicate that the elevation of serum TGF-β1 levels contributes to the protective effect of KRN pretreatment in septic neonates and is mediated through CD1d.

In this study, we observed an early Th1-biased response at 5 h after KRN administration in neonatal mice, with increases in serum levels of IL-2 and frequency of CD69 expression on hepatic iNKT cells, followed at 10 h by an increase in serum IFN-γ levels. At 30 h after KRN administration, on the other hand, hepatic iNKT cell activation returned to baseline, as did levels of the Th1-associated cytokines IL-2, IL-12, and IFN-γ. Most notably increased at this time point was TGF-β1, which is alternately described as a Th2- or Th17-associated cytokine, and possesses many anti-inflammatory properties as outlined above.

Our data show increases in TGF-β1 and IL-6 in the serum of neonatal mice 30 h after KRN administration, as well as in IL-17A and IL-23. This profile is suggestive of a Th17-polarizing milieu. Some LPS-negative α-proteobacteria (e.g., *S. capsulata* and *E. muris)* possess cell wall glycosylceramides that serve as direct targets for iNKT cells, and upon recognition of these targets, iNKT cells are able to facilitate bacterial clearance and control infection (40, 41). It has been speculated that iNKT cells may have evolved to target such bacteria lacking TLR ligands (42). Given that iNKT cells can evoke strong cytokine signaling cascades in the absence of activation by TLR ligands, their activation may thus offer a route for increased Th17 polarization in neonates. Th17 immunity is important for maintaining epithelial barrier function; in neonates with relatively impaired opsonization ability due to complement insufficiency and reliance on maternal antibody transmission, this may be critical in preventing translocation of bacteria and evading lethal infection (43, 44).

It is understood that upon activation iNKT cells present in liver sinusoids arrest and exert various effector functions (13). In contrast, after antigen exposure in the lungs, iNKT cells present there appear to extravasate and exert their effector functions in the surrounding parenchyma, rather than entering the circulation (13). This raises the important question as to whether or not KRN’s effect on pulmonary inflammation and injury in our sepsis model is the result of its action on iNKT cells resident in the lungs or liver, and if iNKT cell populations in different compartments respond similarly to antigen stimulation in the neonate. In light of our experiments demonstrating a correlation between hepatic iNKT cell activation, serum levels of proinflammatory cytokines, and lung injury, it seems likely that this lung injury is the result of systemic IFN-γ release. However, there may be an additional effect of activated iNKT cells within the lungs, and an investigation into the response of iNKT cells within different compartments to KRN administration in the neonate is warranted in the future.

An important limitation of our cytokine array analysis is the use of pooled serum samples. This approach was useful to broadly identify potential mediators in response to iNKT cell activation, and led us to examine the effects of TGF-β1 on septic neonates. Although the change in cytokine levels observed in the array study could not be adequately analyzed statistically to lend itself to a clear conclusion, the results provide guidance for future studies. In addition, we have used a one-way ANOVA to analyze the statistical difference among the four groups including sham, vehicle, 0 h, and −30 h treatment groups in Figure 1. In the future, when we conduct KRN administration at multiple time points surrounding sepsis onset to study the interaction between time and treatment, the sample size of each group will need to increase in order to have sufficient statistical power to perform a two-way ANOVA analysis.

Herein, we have established for the first time the response of neonatal iNKT cells to a CD1d ligand, and explored the effect of activation of neonatal iNKT cells at multiple time points around sepsis induction. We have confirmed that the protective effect of iNKT cell pre-activation is mediated by the CD1d molecule through a systemic increase in TGF-β1. Future research in this area should aim to shed light on differences in iNKT subsets resident in different compartments and on generating pharmacologic agents that may be used to polarize these cells toward a protective phenotype in the setting of neonatal infection.

## Ethics Statement

This study was carried out in accordance with the recommendations of the National Institutes of Health Guidelines for the Use of Laboratory Animals. The protocol was approved by the Animal Care and Use Committee of the Feinstein Institute for Medical Research.

## Author Contributions

Design and concept: AB, W-LY, AS, and PW. Conduct of experiments: AB and LH. Results analysis and interpretation: AB, W-LY, AS, JN, GC, and PW. Manuscript preparation, revisions, and approval: AB, W-LY, and PW.

## Conflict of Interest Statement

The authors declare that the research was conducted in the absence of any commercial or financial relationships that could be construed as a potential conflict of interest. The reviewer SH and handling Editor declared their shared affiliation.

## Supplementary Material

Figure S1 Kinetic analysis of KRN treatment on serum levels of various cytokines in neonatal mice. C57BL/6 pups received KRN (0.2 µg/g BW) i.p. Serum was harvested at 1, 5, 10, 20, and 30 h after KRN administration. Serum samples were pooled (5–10 samples per group) and a multi-analyte ELISArray was performed. Fold change in absorbance at 450 nm compared to baseline control (Ctrl) is shown.

Figure S2 Effect of KRN treatment on serum levels of various cytokines in C57BL/6 WT and CD1d knockout (KO) neonatal mice. C57BL/6 WT and CD1d KO pups received KRN (0.2 µg/g BW) i.p. and serum was harvested 30 h later. Serum samples were pooled (5–10 samples per group) and a multi-analyte ELISArray was performed. Fold change in absorbance at 450 nm compared to baseline control is shown.

## Abbreviations

ANOVA, analysis of variance; α-GalCer, α-galactosylceramide, (*N*-[(*1* *S,2S,3R*)-1-[(α-D-galactopyranosyloxy)methyl]-2,3-dihydroxyheptadecyl]-hexacosanamide); BW, body weight; CS, cecal slurry; DMSO, dimethyl sulfoxide; ELISA, enzyme-linked immunosorbent assay; FSC, forward scatter; FVD, fixability viability dye; H&E, hematoxylin and eosin; ILL, innate-like lymphocyte; iNKT, invariant natural killer T; i.p., intraperitoneal; KC, keratinocyte chemoattractant; KRN, KRN7000, a synthetic α-galactosylceramide analog, ((*2* *S,3S,4R*)-1-*O*-(α-D-galactopyranosyl)-*N*-hexacosanoyl-2-amino-1,3,4-octadecanetriol); MIP-2, macrophage inflammatory protein-2; SSC, side scatter; TLR, toll-like receptor.
