# Supplementary figures and images for: Activation of Invariant Natural Killer T Cells Redirects the Inflammatory Response in Neonatal Sepsis
Source: Front Immunol. 2018 Apr 23;9:833. doi: 10.3389/fimmu.2018.00833 (PMC5922987; doi:10.3389/fimmu.2018.00833)

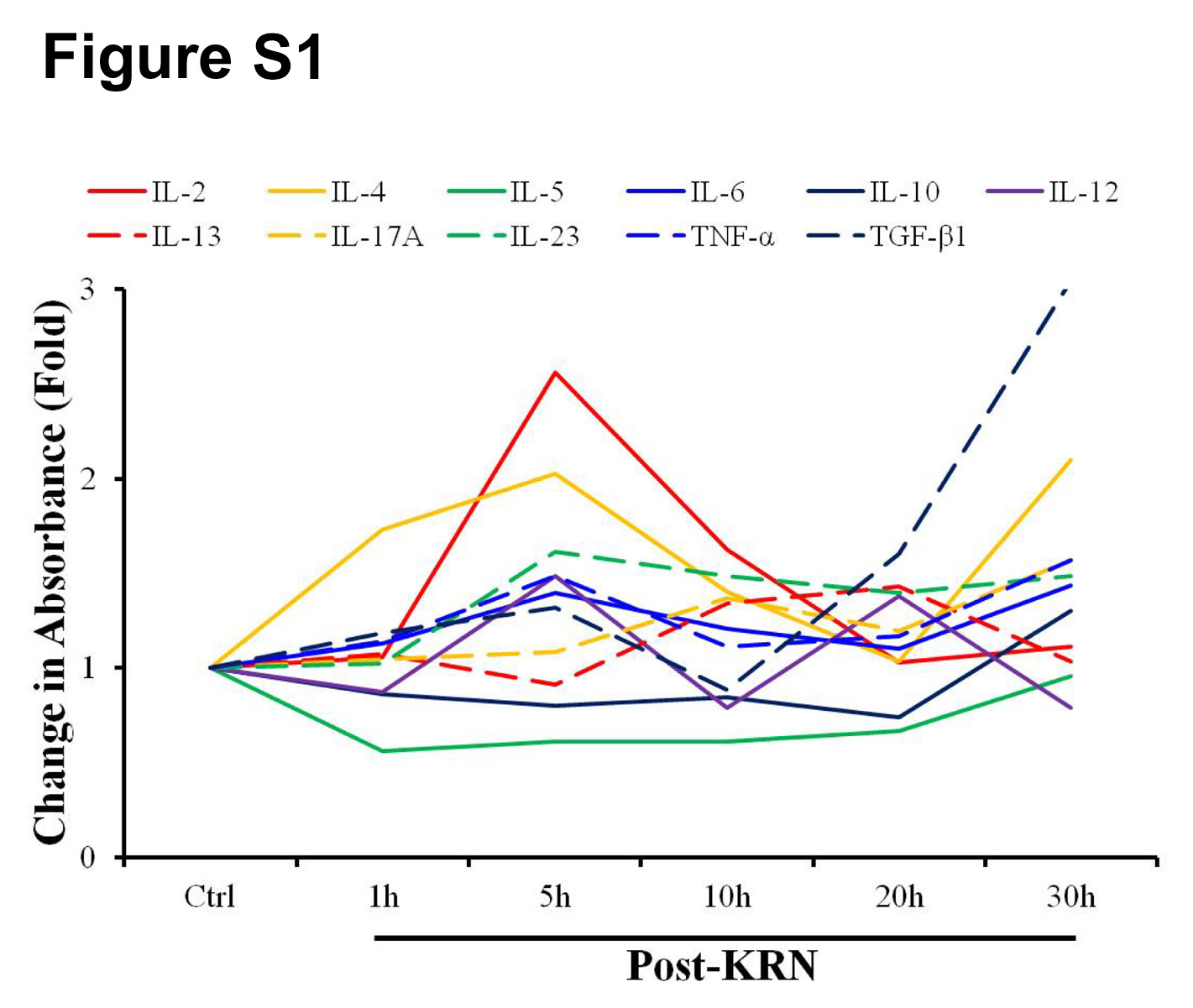

Supplement: Figure S1 — Kinetic analysis of KRN treatment on serum levels of various cytokines in neonatal mice. C57BL/6 pups received KRN (0.2 µg/g BW) i.p. Serum was harvested at 1, 5, 10, 20, and 30 h after KRN administration. Serum samples were pooled (5–10 samples per group) and a multi-analyte ELISArray was performed. Fold change in absorbance at 450 nm compared to baseline control (Ctrl) is shown. [file image_1.jpeg]

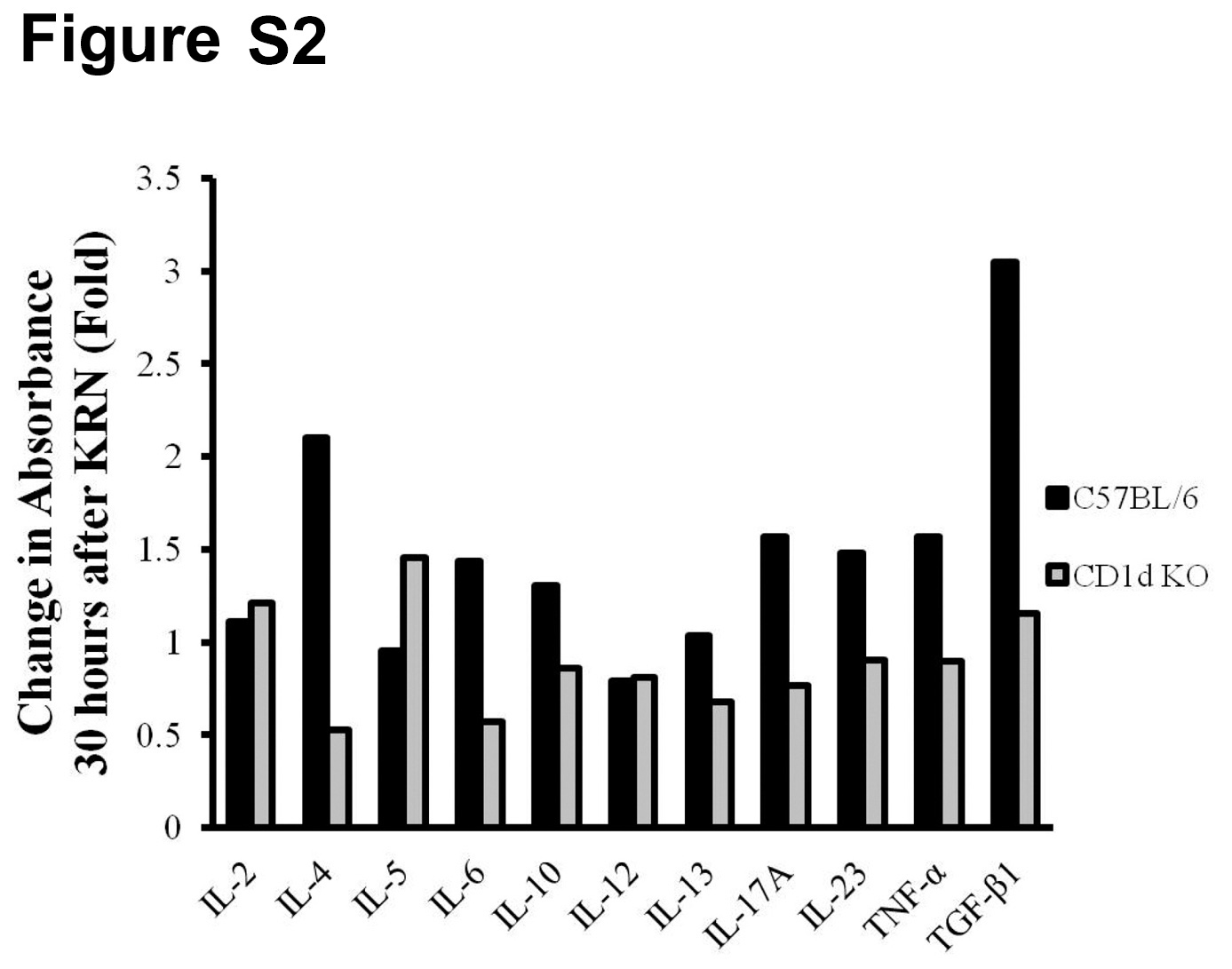

Supplement: Figure S2 — Effect of KRN treatment on serum levels of various cytokines in C57BL/6 WT and CD1d knockout (KO) neonatal mice. C57BL/6 WT and CD1d KO pups received KRN (0.2 µg/g BW) i.p. and serum was harvested 30 h later. Serum samples were pooled (5–10 samples per group) and a multi-analyte ELISArray was performed. Fold change in absorbance at 450 nm compared to baseline control is shown. [file image_2.jpeg]
